# Is unplanned out-of-hospital birth managed by paramedics ‘infrequent’, ‘normal’ and ‘uncomplicated’?

**DOI:** 10.1186/s12884-017-1617-9

**Published:** 2017-12-22

**Authors:** Belinda Flanagan, Bill Lord, Margaret Barnes

**Affiliations:** University of the Sunshine Coast, ML40, Locked bag 4, Maroochydore DC, Qld 4558 Australia

**Keywords:** Paramedic, Child birth, Labour, Birth before arrival (BBA), Intrapartum care

## Abstract

**Background:**

Unplanned out-of-hospital birth is often perceived as precipitate in nature, ‘infrequent’, ‘normal’ and ‘uncomplicated’. However, international studies report unplanned out-of-hospital birth is associated with increased rates of maternal and neonatal morbidity and mortality. This research describes intrapartum, immediate postpartum and neonatal care provided by paramedics in Queensland, Australia. The objectives were to (1) determine the number of cases where the paramedic documented birth or imminent birth during the study period (2) to describe the incidence of births prior to or during paramedic care (3) to detail any risk factors and/or complications recorded by paramedics during these cases, (4) identify paramedic pain management practices for intrapartum care, and (5) to examine the maternal and neonatal outcomes as documented by paramedics.

**Method:**

A retrospective analysis of Queensland Ambulance Service (QAS) de-identified patient care records, generated from clinical case data between the 1st of Jan 2010 and 31st of Dec 2011, was undertaken. Descriptive analysis and *x* ^*2*^ tests were used to test associations between categorical variables, and the Wilcoxon rank-sum for associates between continuous variables which were not normally distributed. Content analysis was utilised to code free text fields.

**Results:**

Six thousand one hundred thirty-five records were identified as intrapartum cases. This represented approximately 0.5% of the annual QAS caseload; 5722 were classified as maternal records and 413 were neonatal records. Paramedics recorded antenatal and/or intrapartum complications in 27.3% (*n* = 1563) of cases. Abnormal maternal vital signs were recorded in 30.1% (*n* = 1725) of cases. Of the 5722 women attended by paramedics during their labour, a birth occured in 10.8% (*n* = 618) of cases. Parity was documented in 41.4% (*n* = 256) of mothers who birthed. Neonatal records were available for 66.8% (*n* = 413) of actual births, 60.0% (*n* = 248) recorded a full set of neonatal vital signs and an Apgar score. When an Apgar score was recorded, 21.8% (*n* = 91) scored ≤7 out of 10.

**Conclusions:**

The research described intrapartum, immediate postpartum and neonatal care provided by paramedics and identified factors that may complicate paramedic clinical management of labouring and birthing women. Further research is required to determine if there are opportunities to improve the paramedic management of such cases.

## Background

Paramedics play an important role in providing intrapartum, immediate postpartum and neonatal care to women in the out-of-hospital environment. The exact nature of this role and the outcomes of such management are not well described in the literature. An axiom expressed by paramedics is that these cases are ‘infrequent’ and often ‘normal’ and ‘uncomplicated’. However, this belief does not reflect existing international literature, and few studies have been published in Australia to support this view. This study therefore aims to determine the frequency of intrapartum, immediate postpartum and neonatal care that is provided by paramedics and to describe the frequency of risk factors and/or complications encountered during these cases.

Various terms are used in the literature to define an unplanned out-of-hospital birth; for example unplanned ‘Birth Before Arrival (BBA)’, or an ‘out-of-hospital emergency birth’ [1–3]. These births differ from planned home-births because they are normally pre-booked to occur in a hospital or birthing centre setting but for some reason have been born prior to arrival at the facility. Three categories of patients in this genre exist; women who require care and transport while in labour, women who birth just prior to or during paramedic care, and the baby, an additional patient when a birth occurs. Paramedics may be involved in the care of a mother, baby or both when an emergency birth or birthing complication initiates a call to the emergency services number.

In Australia, there is little research concerning the management of labour and birth by paramedics. These patients may be perceived as low risk cases that only require transport, or if birthing, considered a precipitate birth that is anecdotally ‘normal’ and ‘uncomplicated’. However, births that occur in unplanned environments are associated with several risks. Without prior knowledge of maternal risk factors the ability of health care providers to identify women at risk of birthing in the unplanned environment is limited, and this may compromise management of the birth [4]_._ International literature indicates that in developed countries, unplanned births before arrival at hospital are predominantly attributed to a lack of antenatal care, a multiparous mother or a mother with a history of precipitate birth or a short second stage of labour [5–7].

Unplanned births that occur in the out-of-hospital environment often feature high rates of perinatal morbidity and mortality, and studies indicate that the complications suffered are largely due to avoidable causes [6, 8–11]_._ Mothers who experience a birth before arrival have demonstrated an increased incidence of postpartum haemorrhage, postnatal anaemia, perineal tears, genitourinary trauma and retained placenta requiring manual removal [3, 6, 11]_._ Babies may present with increased risk features and require admission to neonatal nurseries for reasons including low birth weight, prematurity, hypothermia and hypoglycaemia [3, 4, 11]_._

Studies that have reported cases of women who birth before arrival at hospital found that paramedics attended between 28.2% and 91.5% of all unplanned BBA’s [12]. In order to understand the paramedic role in managing births before arrival at hospital this study aimed to (1) determine the number of cases where the paramedic documented birth or imminent birth, and the incidence of these cases based on the total caseload of emergency calls during the study period (2) to describe the incidence of births prior to or during paramedic care (3) to detail any risk factors and/or complications recorded by paramedics during these cases, (4) identify paramedic pain management practices for intrapartum care, and (5) to examine the maternal and neonatal outcomes as documented by paramedics.

## Methods

### Selection and description of cases

Queensland is the second largest state in Australia with an area of 1,727,000 km^2^. More than half of the population live outside the greater metropolitan area of Brisbane, which is a large proportion compared with the rest of Australia [13]. In the state of Queensland most births occur in a hospital. In 2014, 98% of women gave birth in a hospital; 1.8% occurred in a birthing centre, 0.3% occurred at home and 0.4% in other settings including births occurring before arrival at hospital [14]. The Queensland Ambulance Service (QAS) provides emergency medical care and transport by road ambulance and aircraft for all residents of the state of Queensland (population approx. 4.7 million). The QAS transported approximately 675,000 patients during the study period 2010/11 [15].

QAS paramedics record patient information and treatment provided electronically on a tablet computer using Victorian Ambulance Clinical Information System software (VACIS). All records are stored in a central, electronic data warehouse. The software has options of ‘free-text’ and ‘drop-down lists’ which allows paramedics to document patient medical history, current medications, allergies, chief complaint or clinical findings, and treatment provided. The VACIS system also enables documentation of risk factors associated with each case. An application was made to the Commissioner, Queensland Ambulance Service for access to de-identified patient care records.

Specific inclusion criteria were used to extract data to include in this study (Table 1). The key words were based on the options available in the dropdown lists and free text areas that made reference to intrapartum care. Gestation was undefined and cases identified as a duplicate, inter-facility transfer or non-transport were excluded.

**Table 1:** Inclusion Criteria - Patient care records 1 January 2010 to 31 December 2011

	Key words
Patient complaint	Contractions OR Ruptured Membranes OR Childbirth OR Post-Partum Bleeding OR Breathing Problem/ Difficulty
Comments and Case Description	Contraction OR Labour OR, Childbirth
Secondary Survey	Childbirth (Actual/Labour) OR Contractions AND (Groaning OR Grunting OR Guarding OR Aspiration)
Final Assessment	Childbirth OR Cardiac Arrest OR Febrile OR Deceased OR Altered conscious state

QAS policy requires paramedics to complete a case record for all patients, and when a birth takes place the mother and the newborn should have separate case records. The information included in the case record is entered in specific data fields or is selected from “drop down” menus at the discretion of the paramedic. Fields such as vital signs are mandatory and require an entry to complete the record. However, the paramedic can select a reason that the data was not entered – such as non-compliant patient – and this overrules the mandate. Paramedics are also able to enter free text to describe aspects of the case that include scene findings, medical history or treatment decisions.

### Statistical analysis

The data extracted from the patient record database was manually sorted and coded based on explicit criteria to identify paramedic descriptions of maternal intrapartum complications and information concerning the paramedic management of the case. Using Stata Data Analysis and Statistical software [v13], descriptive statistics were used to describe these outcomes of interest. Chi-squared tests were used to test associations between categorical variables, and the Wilcoxon rank-sum for associates between continuous variables which were not normally distributed. Content analysis was utilised to code free text fields.

## Results

### Incidence

There were 6135 cases which met the inclusion criteria during the period 1 January 2010 to 31 December 2011 (Table 1). This represented approximately 0.5% of the overall annual QAS caseload; 5722 were classified as maternal records and 413 were neonatal records. Women who were reported to be in labour but refused transport by paramedics represented 0.2% (*n* = 13) of the data set; women who birthed but refused transport represented 0.08% (*n* = 5) and 0.08% (*n* = 5) of cases provided no indication of the patient being transported or refusing transport (missing data).

The maternal age range was 13 years to 47 years (mean age of 27.3, median of 27 and interquartile range of 22–32). Age was missing in 1.2% (*n* = 68) of cases. Most intrapartum cases (40.6%, *n* = 2325) occurred in the metropolitan areas of South East Queensland, Australia. This was compared with Queensland Government Birth Statistics which reflected a similar frequency of births in each Queensland Health region (Table 2).

**Table 2:** Comparison of QAS obstetric cases to recorded births for each health service

Location	QAS Obstetric Cases	Percent	Queensland Births	Percent
Metro South	1518	26.5%	29,804	24.4%
Metro North	807	14.1%	23,124	18.9%
Cairns, Hinterland	623	10.9%	7001	5.7%
West Moreton	534	9.3%	7769	6.4%
Townsville	490	8.6%	6724	5.5%
Darling Downs	355	6.2%	7549	6.2%
Gold Coast	321	5.6%	11,746	9.6%
Central Queensland	285	5.0%	6377	5.2%
Sunshine Coast	246	4.3%	7827	6.4%
Wide bay	223	3.9%	4561	3.7%
Mackay	155	2.7%	4952	4.1%
North West	127	2.2%	1255	1.0%
Cape York (Inc. Torres Strait-Northern Peninsula)	18	0.3%	1042	0.9%
South West	16	0.3%	869	0.7%
Central West	4	0.1%	363	0.3%
Interstate/Overseas	–	–	1183	1.0%
Total Cases	5722	100%	122,146	100%

Source: Queensland Government Birth Statistics [14, 15]

The age groups of ≤16 yrs. and ≥35 yrs. were cross-tabulated to geographical area as pregnancies within these age groups are identified in the literature as having an increased possibility (high-risk) of complications such as low birth weight, preterm infants and still-birth [16, 17]. Results revealed that the remote region of Cape York had a low number of cases (*n* = 18), yet 38.9% (*n* = 7) were identified as high-risk age groups.

### Risk factors

An option to record “Risk Factors” within the VACIS system allows paramedics to identify specific risk factors pertinent to each case. While acknowledging that these reporting fields are not mandatory, paramedics reported that 6.8% (*n* = 388) of mothers who required ambulance attendance for intrapartum care had antenatal risk factors. Risk factors that were recorded by paramedics in this subgroup of cases included gestational diabetes (18.3%, *n* = 71), hypertension (17.3%, *n* = 67), smoking (29.6%, *n* = 115), alcohol (3.4%, *n* = 13), illicit drug use (3.1%, *n* = 12), obesity (2.3%, *n* = 9), age (2.1%, *n* = 8) and no antenatal care (24%, *n* = 93). These risk factors were recorded by the paramedic during the clinical history taking process. Although the frequency of recorded risk factors was low, the result indicates that risk-factors are being recorded by paramedics during the episode of care. However, the actual number of cases that involved risk factors cannot be ascertained due to the non-mandatory collection of this data.

### Maternal outcomes

Two areas of documented care were examined to determine maternal outcomes; maternal pain management and maternal vitals signs. These two areas were chosen as they were consistently recorded within the data and mandatory areas of reporting.

The paramedic management of a mother’s pain during the intrapartum period was examined according to the mother’s reported pain score and the nature and type of analgesia administered. Paramedics normally use a 0–10 verbal numeric rating scale (VNRS) to record pain severity and document the score provided by the patient in the electronic patient care record. However, the VACIS system also includes adjective descriptions (or labels) of pain severity against the following VNRS points (2 = mild, 5 = moderate, 8 = severe). It was noted that pain scores were not normally distributed across the population, with a higher frequency of scores associated with the adjective labels on the scale.

During the research period, analgesics available to paramedics included morphine sulphate and methoxyflurane. QAS practice guidelines did not restrict the use of analgesia in labouring mothers and the guidelines do not list administration precautions during the intrapartum period. An initial VNRS was recorded in 4454 (77.8%) of maternal cases, and included 1346 cases (30.2%) where the initial VNRS was recorded as zero – by default the field is blank. The median initial maternal pain score was 2/10. However, it was not possible to determine if a pain score was taken during, prior to or after a contraction. Methoxyflurane was administered to 19.6% (*n* = 1119) of all maternal cases. Morphine was administered to 0.7% (*n* = 40) of mothers, with 0.3% (*n* = 16) receiving both morphine and methoxyflurane**.** Of women who birthed while in paramedic care (*n* = 356), 1.4% (*n* = 5) were administered morphine, 24.4% (*n* = 87) received methoxyflurane and 0.8% (n = 3) received both morphine and methoxyflurane for pain relief. An examination was completed to identify any correlation in opioid analgesia administered and a depressed Apgar score, however the low frequency of neonatal observations did not provide an opportunity to determine any correlation between Apgar and opioid administration.With the exception of 1.0% (*n* = 60) of cases that did not record any maternal observations (classified as missing data), abnormal maternal vital signs were recorded in 30.1% (*n* = 1725) of mothers who required intrapartum and immediate postpartum care. Vital signs identified as abnormal (*n* = 1725) were blood pressure, respiratory rate, temperature, SpO_2_ and blood glucose level (Table 3)*.*

**Table 3:** Abnormal Maternal Observations *(n = 1725)*

(a) Outcome	N=Number identified	Percentage
Hypertensive (≥140/90)	1059	61.4%
Fever (>37.8 °C)	14	0.8%
Respirations (>24/min)	539	31.3%
SpO2 (<97%)	0 (none were recorded)	0%
BSL (<4)	26	1.5%
BSL (>7)	87	5.0%

(a) Reporting of multiple abnormal observations was permitted, so numbers cannot be cumulated

According to the Royal College of Obstetricians and Gynaecologists [18], mild hypertension in pregnancy is classified as a blood pressure greater than 140/90 mmHg and severe hypertension reported as blood pressure greater than 170/110 mmHg. Mild hypertension was identified in 18.5% (*n* = 1059) of the total maternal cases, with 8.1% (*n* = 463) documented as having a blood pressure in the severely hypertensive category. An elevated maternal temperature was recorded in 0.2% (*n* = 14) of all investigated cases; the reason for this could not be determined from the case notes.

### Intrapartum care

A birth was reported in 10.8% (*n* = 618) of cases. Of these, 42.4% (*n* = 262) occurred prior to ambulance arrival and 57.6% (*n* = 356) occurred in paramedic care. Parity was documented in 41.4% (*n* = 256) of mothers who birthed, 58.6% (*n* = 362) of the case records did not identify parity. Mothers who birthed were more likely to be multigravida women (86.3%, *n* = 221) with primigravida identified in a smaller number of cases (13.7%, *n* = 35). Complications of labouring women and those who went on to birth while in paramedic care were examined to identify the frequency of complications recorded by paramedics (Table 4)*.* Due to the potential for multiple problems only those described in the QAS paramedic clinical practice guidelines were examined.

**Table 4:** Intrapartum Complications identified

	Labouring women *(n = 1543)*		Women who also birthed *(n = 127)*	
Complication (a)	N = Number identified	Percentage	N = Number identified	Percentage
Antepartum haemorrhage	163	10.6%	13	10.2%
Cord prolapse	10	0.7%	1	0.8%
Placenta Previa	48	3.1%	1	0.8%
Multiples (twins or triplets)	99	6.4%	14	11.0%
Premature (20wks < 36 + 6wks)	836	54.2%	36	28.4%
Breech	105	6.8%	9	7.1%
Stillbirth	–	–	13	10.2%
Threatened Miscarriage (<20wks)	80	5.2%	40	31.5%

(a) Reporting of multiple complications was permitted, so numbers cannot be cumulated

Complications that were reported in small numbers and were not included in the analysis included maternal HIV, Hep C, cervical stitch in-situ, Streptococcus or Cytomegalovirus, Chorioamnionitis, Bicornuate uterus, Polyhydramnios, Oligohydramnios, Intrauterine Growth Restriction, congenital abnormalities of the fetus and pre-existing maternal comorbidities.

Paramedics recorded that 2.4% (*n* = 15) of women who birthed before arrival stated that they had been sent home from hospital within the 12 h prior to calling the ambulance service due to being in either a ‘false labour’ or ‘early labour’ that did not yet require hospital admission. It should be noted that this advice could also have been provided to other women as this is not a mandatory area of reporting. Mothers transported from a planned homebirth (0.4%, *n* = 24), included those that were classified as a ‘failure to progress in second stage’, ‘maternal fatigue’, ‘post-partum haemorrhage’ or a ‘retained placenta’.

Third stage or birth of the placenta was recorded in 20.9% (*n* = 129) of cases, however 54.5% (*n* = 337) did not record the management or status of third stage as it is not mandatory. Third stage was coded based on whether a placenta was birthed or not. Records did not enable reliable reporting of assessment of the integrity of the placenta. When paramedics did record the birth of the placenta, 31% (*n* = 40) recorded fundal massage. However, it was noted in cases where the placenta had not been birthed (*n* = 152) that fundal massage was initiated by paramedics in 19.7% (*n* = 30) of these cases despite no evidence of postpartum haemorrhage in the documentation and documentation of normal maternal vital signs. Based on the reported observations the use of fundal massage in these cases appears to be inconsistent with evidence-based recommendations. Paramedics documented a postpartum haemorrhage (blood loss >500 ml) in 6.2% (*n* = 38) of cases, however the cause was unknown as in most cases paramedics did not record this finding.

### Neonatal outcomes

When a birth occurred, information regarding newborn care was derived from the ‘Case Description’ field of the maternal records and the newborn case records. Queensland Ambulance Service Policy states all neonates must have a separate case record as they are classified as a second patient after a birth. This only occurred in 66.8% (*n* = 413) of cases.

All newborn patient care records (100%) documented either an Apgar score (normally 1 min and 5 min after the birth), or heart rate or respiratory rate. However, only 60.0% (*n* = 248) recorded continuing vital signs in addition to the initial Apgar score. When an initial Apgar score was documented (62.8%, *n* = 388), 21.8% (*n* = 91) of newborns scored ≤7 and 49.9% (*n* = 308) scored ≥8, 37.2% (*n* = 230) of births did not record an Apgar. When the birth was premature, less than 37 weeks gestation (28.4%, *n* = 36), 13.9% (*n* = 5) of premature neonates had an Apgar ≤7.

Table 5 examines the frequency of abnormal observations in newborns. As newborns can take time to adjust to extra-uterine life; both the initial and final observations were examined when documented.

**Table 5:** Abnormal Neonatal Observations *(Initial n = 224, Final n = 192)*

	Initial Observations		Final Observations	
Observation (a)	N = Number	%	N = Number	%
Heart Rate (<110)	21	9.4%	22	11.5%
Heart Rate (>160)	24	10.7%	23	12%
Temperature (≤36.2 °C)	15	6.7%	15	7.8%
Respiratory rate (<40)	162	72.3%	131	68.2%
Respiratory Rate (>60)	2	0.9	1	0.5%
Blood Glucose level (<2.6 mm/l)	0	0%	0	0%

(a) Reporting of multiple observations was permitted, so numbers cannot be cumulated

Newborns requiring resuscitation (4.5%, *n* = 27) included those that received suction and ventilation by artificial means or those that required resuscitation including cardiopulmonary resuscitation. These events were cross-referenced when an intra-partum complication was identified (29.6%, *n* = 8) or antenatal risk factor (18.5%, *n* = 5). In all cases the underlying cause for resuscitation could not be determined with the information provided in the documentation.

## Discussion

These results indicate that paramedic involvement in intrapartum and immediate postpartum care in Queensland represent a small percentage of the overall ambulance service workload. In addition, the results revealed that, although complications associated with the care of labouring and birthing women are low in this study population, complications can be challenging to manage when occurring in the community setting, particularly if the setting is a distance from specialist care.

This study identified antenatal and intrapartum risk factors that are known to increase the risk of pregnancy complications and possibly have adverse effects on maternal and foetal outcome. Maternal age was documented by paramedics as a risk factor in pregnancy, although QAS guidelines do not specify when age becomes a risk factor, only referring to ‘Advanced Maternal Age’, the age groups of <16 years and >40 years were documented by paramedics as a ‘risk’ and this is supported by other areas of research [17, 19, 20]. Teenagers (<16 years) have been shown to have increased rates of ectopic pregnancy, pre-eclampsia, eclampsia, preterm labour and premature rupture of membranes [21, 22], and current evidence suggests advanced maternal age (>35 years) increases the risk of miscarriage, ectopic pregnancy and stillbirth [16, 17]. Studies also report elevated maternal morbidity resulting from hypertensive disorders and gestational diabetes in this category of patient [17]. Both conditions may predispose the mother and baby to health problems that require specialist care and impose an increased risk of complications during the intrapartum and immediate postpartum period [23, 24].

The consequences of antenatal hazards are well known, with behaviours such as smoking, illicit drug use and alcohol consumption increasing the odds of conditions such as miscarriage, ectopic pregnancy, placental abruption, preterm birth and low birth weight babies [25–27]. In this study, an additional antenatal risk factor identified by paramedics was a lack of antenatal care. Research suggests the implications associated with no antenatal care in the out-of-hospital environment are also associated with poor maternal and neonatal health outcomes [1, 28].

Studies designed to profile women who do not access antenatal care show that the most common barriers to attendance are maternal age, multiparity, low socioeconomic status and unmarried status [29–33]. When a mother does not receive antenatal care and has no information regarding the health of the pregnancy, the management of the case may become complex. Information regarding the position of the baby and placental position are essential to reduce the risk of adverse outcomes. Abdominal palpation and assessing foetal heart sounds are not normally skills performed by paramedics, and conditions such as placenta praevia would be concealed if no antenatal care was accessed by the mother. The consequences of such complications may impact a paramedics descision making with regard to urgency of transport and choice of definitive care.

Mothers who present with abnormal vital signs during the intra-partum period may be suffering from conditions that could complicate the birth and the health of the newborn. Maternal illness and adverse vital signs are not only detrimental to the mother but may also impact foetal development, cause foetal distress or foetal death and in some cases impact early childhood growth and development [34]. These conditions may go undetected by paramedics unless they are aware of normal maternal vital sign values in pregnancy, how pregnancy changes the normal physiology of the mother and how abnormal vital signs can become life threatening unless managed appropriately.

Research has shown that hypertensive disorders for example are associated with high levels of both maternal and foetal morbidity and mortality [35]. An elevated blood pressure during labour can contribute to placental insufficiency and subsequent foetal hypoxia [36] and has been shown to increase the incidence of postpartum preeclampsia [37]. An elevated blood pressure (>140/90) was identified in this data set however the mode of measurement was not documented. Automatic blood pressure cuffs are used often by paramedics in clinical practice, however they are not recommended for antenatal use in other clinical settings. Research suggests that by using automatic blood pressure cuffs systolic blood pressure can be underestimated by up to 10-20 mmHg [38–40]. It is important within paramedic practice to establish clear guidelines that recommend the use of manual blood pressure cuffs when establishing a base line set of maternal vital signs.

When a birth occurred there was often limited documentation in this data set concerning the clinical findings or actual management of the birth itself. In addition, the management of third stage or birth of the placenta identified interventions that were inconsistent with evidence-based guidelines on the basis of the documented information. For example, several cases documented fundal massage prior to the birth of the placenta. This is known to be associated with uneven separation of the placenta from the fundus and could contribute to excessive maternal blood loss [41, 42]_._

The third stage of labour is defined as the moment the baby is born until the expulsion of the placenta, cord and membranes from the uterus. Third stage management is commonly referred to as ‘active’, ‘expectant’ or ‘physiological’. The term active management of third stage has various meanings but commonly refers to the use of uterotonic drugs immediately following the birth of the baby, controlled cord traction and early clamping and cutting of the cord, whereas expectant or physiological third stage involves allowing the placenta to deliver spontaneously or aided by gravity [43, 44].

As there was no QAS clinical guideline at the time for the management of third stage and no uterotonic medications available for use by paramedics in Queensland, paramedics utilised expectant or physiological management of third stage. The Royal Australian and New Zealand College of Obstetricians and Gynaecologists [41] and the World Health Organisation [42] both recommend active management as the most appropriate form of management for the third stage of labour by ‘skilled attendants’. They state that the routine use of prophylactic oxytocics, cord-clamping and controlled cord traction should be available to all women as it has been shown to reduce the frequency of PPH by up to 50% [41].

As state ambulance service guidelines throughout Australia differ in their recommendations for the management of third stage, it is essential to achieve consensus and consistency in practice using highest available evidence. Further research is required to examine the most appropriate method of third stage management or at least the availability of oxytocic drugs in the event of a postpartum haemorrhage in the out-of-hospital setting.

Various studies acknowledge that newborns birthed in the out-of-hospital environment have a high rate of morbidity and mortality and the complications are largely due to preventable causes [8, 9, 11].

Studies relate high perinatal mortality to prematurity and low birth weight with hypothermia as the most common complication recorded. The research identifying hypothermic babies born in the out-of-hospital environment relies on the temperature once presenting to definitive care. Of the 618 babies birthed in this study only 15 had a temperature taken, all were classified as hypothermic (<36.2 °C). However, these results are inconclusive because of the method of temperature taking, which is associated with error. All ambulance vehicles in Queensland carry tympanic thermometers. Tympanic thermometers are contraindicated in the use of newborns because of the vernix in the ears at birth; this prevents the infrared probe accessing the tympanic membrane which may be associated with an inaccurate temperature reading [45]. The temperatures taken in this study were done so by a tympanic method, therefore the temperatures recorded may not be correlated with core temperature. Premature labour or birth is particularly complicated by neonatal hypothermia and was identified in 14.6% (*n* = 836) of cases. The QAS recommendation for the out-of-hospital care of a premature infant recommends wrapping the baby in cling-wrap or plastic-film with the head exposed without drying beforehand [46], although this was not recorded as a form of management by paramedics in this study. This method, in combination with skin-to-skin contact, has been shown to assist with the thermoregulation of the premature infant after birth and help prevent hypothermia [47].

Neonatal oxygen saturation levels were not able to be recorded in any of the neonatal records. However, neonates were reported to having received ventilation and oxygen administration. At the time of this study Queensland paramedics did not have paediatric probes to measure oxygen saturations levels in neonates. According to the Australian Resuscitation Council Neonatal Resuscitation Guidelines, “oximetry is recommended when the need for resuscitation is anticipated, when positive pressure is administered for more than a few breaths, when persistent cyanosis is suspected” [48]. QAS Neonatal Resuscitation Guidelines [46] state ‘pulse oximetry may assist during resuscitation’ and outline expected SpO2 levels after birth. QAS confirmed that until new equipment is purchased (to be introduced in December 2014) only adult SpO2 probes are available to paramedics. Further investigation is required to examine the use of pulse oximetry during neonatal resuscitation by paramedics.

Since intrapartum cases represent a small percentage of the annual emergency caseload, paramedics have infrequent exposure to a birth situation. Studies indicate that caring for pregnant and birthing women is an area in which paramedics feel less prepared and may lack confidence as it constitutes a minor component of their education and overall case load [49]. Due to a high demand for clinical placements in hospitals and birth centres and competition with midwifery and medical students requiring clinical placement in Queensland hospitals [50], most paramedic students have very little exposure to obstetrics during their initial training [51]. Although this differs between the various entry-to-practice paramedic undergraduate programs in Australia, obstetrics related content generally occupies only a minor component of paramedic curriculum.

While many births occur without complication, paramedics must be able to identify and accurately manage pregnancy-related conditions using evidence-based guidelines to provide safe and effective care of mothers and babies. In addition, due to the low level of exposure to obstetric cases, and the safety consequences associated with managing high risk events such as postpartum haemorrhage, maintenance of the paramedic’s knowledge and skills is a challenge that requires further investigation to identify means of preparing paramedics to deal with these low frequency cases.

### Limitations and recommendations

Every effort was made to capture all possible keywords to locate all intrapartum cases attended in the study period, however, it is acknowledged that cases may be coded in error by paramedics and therefore may not be inclded in the dataset. Information concerning the management of birth, specifically interventions performed by paramedics on scene and during transport was not consistently documented. Some babies did not receive an individual case record that would allow for a comprehensive picture of neonatal outcome. Information concerning neonatal outcome for those missing records appeared within the maternal case record. This may indicate a lack of adherence to organisation policy. As a result, missing data is a limitation of this study.

## Conclusion

This research acknowledges that this population, although a small proportion of paramedic caseload, can be high-risk and possess factors that may complicate paramedic clinical management. Although unplanned births that occur in the out-of-hospital environment account for less than 1% of all Queensland births, they are associated with considerable perinatal mortality and morbidity. While many births that occur in paramedic care are uncomplicated, paramedics are expected to use evidence-based guidelines to identify, manage and refer patients with high-risk conditions or emerging problems related to pregnancy, recognise and manage a severely compromised pregnant patient and provide appropriate care during the intrapartum and post-partum period. For this to be possible, obstetric content within paramedic curriculum and in-service education provided to paramedics should comprehensively address these infrequent but potentially ‘high risk’ patients.

## Abbreviations

BBA: Birth before arrival
QAS: Queensland ambulamce service
VACIS: Victorian ambulance clinical information system software
VNRS: Verbal numeric rating scale
